# Preoperative co-application of bevacizumab and tissue plasminogen activator in vitrectomy for proliferative diabetic retinopathy

**DOI:** 10.5935/0004-2749.2023-0001

**Published:** 2024-02-23

**Authors:** Cengiz Aras, Fevzi Senturk, Sevil Karaman Erdur, Mehmet Selim Kocabora, Mahmut Dogramaci, Cahit Burke, Efe Aras

**Affiliations:** 1 Department of Ophthalmology, Medicine Faculty, Istanbul Medipol University, Istanbul, Turkey; 2 Department of Ophthalmology, The Princess Alexandra Hospital NHS Trust, London, UK; 3 Medicine Faculty, Yeditepe University, Istanbul, Turkey

**Keywords:** Diabetic retinopathy, Bevacizumab, Plasminogen activators, Vitrectomy, Operative time

## Abstract

**Purpose:**

To investigate the clinical benefits of the co-application of bevacizumab and tissue plasminogen activator as adjuncts in the surgical treatment of proliferative diabetic retinopathy.

**Methods:**

Patients who underwent vitrectomy for proliferative dia-betic retinopathy complications were preoperatively given in-travitreal injection with either bevacizumab and tissue plasminogen activator (Group 1) or bevacizumab alone (Group 2). Primary outcomes were surgery time and number of intraoperative iatrogenic retinal breaks. Secondary outcomes included changes in the best-corrected visual acuity and postoperative complications at 3 months postoperatively.

**Results:**

The mean surgery time in Group 1 (52.95 ± 5.90 min) was significantly shorter than that in Group 2 (79.61 ± 12.63 min) (p<0.001). The mean number of iatrogenic retinal breaks was 0.50 ± 0.59 (0-2) in Group 1 and 2.00 ± 0.83 (0-3) in Group 2 (p<0.001). The best-corrected visual acuity significantly improved in both groups (p<0.001). One eye in each group developed retinal detachment.

**Conclusion:**

Preoperative co-application of bevacizumab and tissue plasminogen activator as adjuncts in the surgical treatment of proliferative diabetic retinopathy shortens the surgery time and reduces the number of intraoperative iatrogenic retinal breaks.

## INTRODUCTION

Vitreous surgery for the treatment of proliferative diabetic retinopathy (PDR) aims to remove vitreous hemorrhage, release vitreoretinal tractions and retinal reattachment, and treat retinal ischemia. The major concerns for these cases are missed posterior hyaloid remnants caused by vitreoschisis, intraoperative, and postoperative hemorrhage, retinal break formation, reproliferation, and retinal re-detachment^(1,2)^.

Bevacizumab is a recombinant monoclonal antibody against vascular endothelial growth factor (VEGF), a major cytokine involved in ocular neovascularization^(3)^. The preoperative injection of bevacizumab has been widely used for >10 years as an adjunct for the regression of neovascular vessels to prevent intraand postoperative hemorrhagic complications in patients with PDR^(4,5)^.

Recombinant tissue plasminogen activator (t-PA), which catalyzes the conversion of plasminogen to plasmin, was previously shown to be effective in inducing posterior vitreous detachment in patients with PDR and vitreous hemorrhage due to diabetic retinopathy^(6)^. The preoperative injection of 25 µg of t-PA was previously used to improve the surgical results of vitrectomy in patients with PDR, but it was ineffective^(7,8)^.

In this study, we investigated the clinical efficacy of the preoperative injection of bevacizumab and t-PA on the possible weakening effect of t-PA on adherence to the vitreoretinal interface and improvement of surgical outcomes in vitrectomy for PDR complications.

## METHODS

In this study, a prospective, randomized, single-masked, comparative analysis was performed at Istanbul Medipol University Hospital, Turkey, a tertiary referral center. This study was approved by the Medical Ethical Committee of Istanbul Medipol University and followed the tenets of the Declaration of Helsinki (Decision no. 10840098-772.02-E.58393). Written informed consent was obtained from the patients or their guardians before the procedures.

This study included 43 eyes from 40 consecutive patients with PDR who were scheduled to undergo vitrectomy between June 2020 and December 2021.

The eyes that were planned for vitrectomy for PDR complications were numbered from 1 to 43. Eyes with an odd number were allocated to Group 1 (preoperative bevacizumab and t-PA), and eyes with an even number were allocated to Group 2 (preoperative bevacizumab alone). Vitreous hemorrhage density was graded as mild, moderate, or severe. A severe grade was defined as a dense hemorrhage, completely preventing observation of the fundus. All patients underwent preoperative B-scan ultrasonography or color fundus photography and optical coherence tomography examinations as routinely performed in our clinic.

In both groups, patient characteristics including age, sex, visual acuity, type of diabetes mellitus, history of laser photocoagulation and intravitreal injection of any drug, presence of rubeosis iridis at predilatation, lens status, intraocular pressure, and presence and severity of intravitreal hemorrhage are shown in table 1.

**Table 1:** Baseline characteristics of the patient population

	Group 1	Group 2	p-value
No. of eyes	22	21	-
Sex: F/M [no.]	11/11	12/6	0.289^a^
Age: mean ± SD (range)	53.77 ± 12.15 (23-71)	49.38 ± 13.07 (26-69)	0.280^b^
Laterality: right/left	12/10	10/11	0.482 ^a^
DM: type 1/type 2 [no.]	6/16	7/11	0.358 ^a^
DM duration [years]	16.79 ± 5.01	16.01 ± 5.69	0.421^c^
Follow-up time(months) (range)	7.68 ± 2.29 (4-11)	7.04 ± 3.42 (3-12)	0.478^c^
Laser treatment: +/- [no.]	7/15	6/15	0.817^a^
Rubeosis iridis: +/- [no.]	2/20	5/16	0.191^a^
Lens: phakic/pseudophakic [no.]	19/3	12/9	0.033^a^
Grade of IVH (preop.)			
Absent	0	0	0.745^a^
Mild	13	10	
Moderate	6	7	
Severe	3	4	
Indications for PPV			0.521^a^
Fibrovascular membrane	16	13	
Tractional RD	6	8	
F/M= female/male; DM= diabetes mellitus; IVH= intravitreal hemorrhage; RD= retinal detachment. ^a^ Pearson chi-square test. ^b^ Student t-test. ^c^ Mann-Whitney U-test.			

All patients in both groups were diagnosed with PDR and showed indications for vitreoretinal surgery because of tractional, thick fibrovascular membrane formation that damaged the macula and optic nerve head and tractional retinal detachment involving or threatening the macula. All cases had varying amounts of hemorrhage in the vitreous cavity. Eyes of patients with diabetes who had complications unrelated to PDR, such as epiretinal membrane, chronic macular edema, and vitreomacular traction, or who had a history of retinal vascular occlu-sive disorders, optic neuropathy, and prior vitreous surgery and eyes with combined tractional and rhegmatogenous detachment were excluded from the study.

Patients were prepared in the usual setting for any intravitreal injection, including 3 min of 5% povidone-iodine application, draping, and lid speculum. After anterior-chamber paracentesis of 0.1 aqueous humor, 1.25 mg/0.05 mL of bevacizumab (Altuzan 100 mg, Roche, Mannheim, Germany) and 0.05 mL of 50 µg t-PA (Actylise 10 mg; Boehringer Ingelheim, Ingelheim am Rhein, Germany) were injected subsequently in the superior quadrant, 3 mm behind the limbus, using a 30-G needle under topical anesthesia. Topical antibiotics were administered until the day of the surgery. Patients were unmasked.

The surgical procedures were similar between the two groups. A single experienced surgeon (CA) performed the surgery on all the patients. The patients underwent preoperative intravitreal injection of bevacizumab with t-PA or bevacizumab alone 3 days before surgery and underwent 23-G, three-port pars plana vitrectomy (PPV). In patients aged >50 years and/or with lens opacification >grade 2, PPV was combined with phacoemulsification and intraocular lens implantation as per the preference of the surgeon. After sufficient clea-rance of the vitreous hemorrhage, surgery continued based on the status of the posterior hyaloid. If the vitreous was detached at the mid-periphery, truncation of the posterior hyaloid was followed by unior bimanual dissection of the preretinal membranes using delamination and segmentation techniques from posterior to anterior. The vitreous base was carefully shaved using a contact or noncontact wide-angle viewing system. Laser photocoagulation was performed on any site of the retina that was previously untreated.

Hemostasis was maintained by raising the infusion pressure or using endolaser photocoagulation or diathermy. All eyes were left with endo-tamponading agents such as air, gas, or silicone oil of 1000 centistokes. Patients were examined on day 1, week 1, and months 1-3 postoperatively. Silicone oil removal surgery was performed in both groups.

The primary outcomes were surgery time including cataract surgery and number of intraoperative iatrogenic retinal breaks. The secondary outcomes included changes in the best-corrected visual acuity (BCVA) and complications 3 months postoperatively.

For statistical analysis, data on visual acuity taken with the Snellen chart were converted to logarithm of minimum angle of resolution (LogMAR) equivalents. The normality between samples was assessed by the Kolmogorov-Smirnov test. Data distribution was not normal. The results were analyzed using the Pearson chi-square test, Fisher’s exact test, Mann-Whitney U-test, and Wilcoxon test (IBM SPSS version 26, IBM Corp., Armonk, NY, USA). A p<0.05 was considered statistically significant.

## RESULTS

A total of 22 eyes from 22 patients (Group 1) who underwent vitrectomy and a pretreatment intravitreal injection with bevacizumab and t-PA injection were compared with 21 eyes from 18 patients (Group 2) who underwent vitrectomy and a pretreatment injection of only bevacizumab. The baseline characteristics of the two groups are presented and compared in Table 1. Few differences in the baseline characteristics of the two groups were noted.

The indications for vitrectomy were thick fibrovascular membranes involving or threatening the macula and/or optic nerve head (n=16 eyes in Group 1; n=13 in Group 2), tractional retinal detachment involving or threatening the macula (n=6 in Group 1; n=8 in Group 2). All cases had varying degrees of intravitreal hemorrhage and attached posterior hyaloid vitreous.

The mean surgery time in group 1 (52.95 ± 5.90 min) was significantly shorter than that in Group 2 (79.61 ± 12.63 min) (p<0.001). The mean number of iatrogenic retinal breaks was 0.50 ± 0.59 (range, 0-2) in Group 1 and 2.00 ± 0.83 (range, 0-3) in Group 2 (p<0.001) (Table 2).

**Table 2:** Mean duration of surgery and number of iatrogenic retinal breaks in both groups

	Primary outcomes		
	Group 1	Group 2	p-value
Mean duration of surgery (min) (range)	52.95 ± 5.90 (45 to 70)	79.61 ± 12.63 (55 to 100)	<0.001^b^
Mean number of retinal breaks (range)	0.5 ± 0.59 (0 to 2)	2.00 ± 0.83 (0 to 3)	<0.001^b^
^b=^ Mann-Whitney U-test.			

Preoperative LogMAR visual acuity values were 1.44 ± 0.11 in Group 1 and 1.40 ± 0.08 in Group 2. At 3 months postoperatively, they were 0.48 ± 0.37 (range, 0.1-1.3) LogMAR in Group 1 and 0.60 ± 0.35 (range, 0.1-1.4) LogMAR in Group 2. Visual acuity improved significantly in both groups (p<0.001; Table 3).

**Table 3:** Preand postoperative 3 months visual acuity [logMAR] (mean ± SD)

	Visual acuity [logMAR]		
	Preoperative mean (range)	Postoperative 3 months mean (range)	p-value
Group 1	1.44 ± 0.11 (1 to 1.50)	0.48 ± 0.37 (0.10 to 1.30)	<0.001^b^
Group 2	1.40 ± 0.08 (1.30 to 1.50)	0.60 ± 0.35 (0.10 to 1.40)	<0.001^b^
^b=^ Wilcoxon test.			

PPV was combined with cataract extraction with phacoemulsification and intraocular lens implantation in 17 and 12 eyes in Groups 1 and 2, respectively. The postoperative endo-tamponading agents were air in 8 eyes, SF6 gas in 7 eyes, and silicone oil in 7 eyes in Group 1. In Group 2, these were air in 2 eyes, SF6 gas in 2 eyes, and silicone oil in 17 eyes (Table 4). This difference was statistically significant between the two groups (p=0.003).

**Table 4:** Distribution of endo-tamponading agents in both groups

	Tamponading agent		
	Air	SF6 gas	Silicone oil
Group 1	8	7	7
Group 2	2	2	17
Fisher’s exact test, p=0.003.			

Silicone oil was removed from all the eyes in both groups. The mean times interval between primary vitrectomy and silicone oil removal was 9.06 ± 1.18 and 9.00 ± 1.10 weeks in Groups 1 and 2, respectively (p=0.29). The mean follow-up time was 7.68 ± 2.29 (range, 4-11) months in group 1 and 7.04 ± 3.42 (range, 3-12) months ranging from 3 to 12 in Group 2 (p=0.478).

Three eyes in Group 1 and four eyes in Group 2 had a postoperative early intravitreal mild hemorrhage. In Group 2, epiretinal membrane developed in 2 eyes, and a macular hole developed in one eye. These membranes were removed during the silicone oil removal surgery. In each group, retinal detachment occurred in one eye. A redo vitrectomy was performed in these eyes, and retinal reattachment was achieved in both eyes.

## DISCUSSION

Most surgical technical problems associated with vitrectomy for patients with PDR are related to neovascularization, firm adherence of retinal membranes, and missed vitreous and posterior hyaloid remnants^(1,2)^. The removal of firmly adherent fibrovascular membranes from the retina increases the risk of intraoperative hemorrhage, retinal break formation, and failure of surgical intervention. Given the risk for vitreoschisis, which was reported to develop in 67.5% of PDR cases^(9)^, surgeons may have difficulty in finding the correct cleavage between the vitreous cortex and the inner limiting membrane. Missed remnants of the hyaloid or vitreous humor may increase the risk of reproliferation after surgery. Posterior vitreous detachment status is a predictive factor of vitrectomy outcomes in patients with diabetic vitreous hemorrhage^(10)^.

Preoperative intravitreal bevacizumab injection before vitrectomy for PDR complications has been routinely employed to prevent neovascular vessel-related surgical complications such as intraand postoperative hemorrhage for more than 10 years^(4,5)^.

Recombinant t-PA catalyzes the conversion of plasminogen to plasmin and is effective in inducing posterior vitreous detachment in patients with PDR and vitreous hemorrhage due to diabetic retinopathy^(6)^. In addition, it has been used to improve the surgical out-co-mes of vitrectomy in patients with PDR^(7,8)^. However, a double-masked multicenter trial on the efficiency of t-PA-assisted vitrectomy in patients with PDR was unable to demonstrate a statistical benefit related to 25-µg t-PA injection 15 min preoperatively. The authors speculated that the possible reasons for failure could be the variability of the action of t-PA, conditional response on the presence of plasminogen, insufficient t-PA dose, and short time interval between the injection and surgery^(8)^. Recently, Falavarjani et al. showed that t-PA injection 5-7 days before surgery is beneficial in decrea-sing the number of iatrogenic retinal break formations and surgery time in cases with diabetic tractional retinal detachment^(11)^.

In this study, bevacizumab and 50 µg of t-PA were coadministered 3 days before surgery to account for the possible weakening effect of t-PA on vitreoretinal adhesion in patients with PDR who were scheduled for vitrectomy. Intraoperatively, we expected to facilitate the separation of the posterior hyaloid, removal of membranes and hyaloid remnants due to vitreoschisis, and decrease the possibility of iatrogenic break formation, which was reported to occur in 60% of vitrectomies for diabetic tractional retinal detachment^(1)^. All patients had one or more PDR-associated complications. We compared the results with those of patients who underwent vitrectomy pretreated with intravitreal bevacizumab injection alone. The surgery time and number of iatrogenic retinal break formations in Group 1 were significantly less than those in Group 2. Short-term tamponades were more frequently used in patients pretreated with intravitreal injections of bevacizumab and t-PA. No statistically significant difference was found in the improvement in visual acuity between the two groups.

Hesse et al. reported the induction of posterior vitreous detachment in 7 of 10 patients with PDR 3 days after t-PA injection^(6)^. We injected bevacizumab and t-PA 3 days before surgery in patients with PDR. In this study, the longer time interval between injection and operation and the presence of varying amounts of blood in the vitreous cavity as a source of plasminogen might have induced the generation of more plasmin enzymes^(12)^. A 3-day interval before surgery is also relatively safe for avoiding possible crunch syndrome due to intravitreal injection of bevacizumab in cases with PDR^(5)^. In addition, we used 50 µg of t-PA, which was greater than the 25 µg used in previous studies, to generate more plasmin. Falavarjani et al. also used 50 µg of t-PA and injected t-PA 5-7 days preoperatively^(11)^.

Previous studies on the treatment of subretinal hemorrhage have proven the safety of the co-application of bevacizumab and t-PA^(13,14)^. However, the co-application of bevacizumab and t-PA does not compromise the anti-VEGF effect of bevacizumab. Bevacizumab is not cleaved by t-PA or t-PA-generated plasmin^(14)^.

Although a single surgeon performed the surgery in all our cases, the limitations of this comparative study were the small number of cases and possible biased conclusions due to the heterogeneity of tractional membranes and detachments in the eyes of both groups. In addition, the study was limited by the lack of multimodal imaging including the absence of intraoperative optical coherence tomography to confirm iatrogenic retinal breaks. Nevertheless, a prospective randomized, comparative analysis was performed in this study, and the novelty and scientific rationality of the approach are its strengths.

In conclusion, this study shows that intravitreal injection of bevacizumab and 50 µg of t-PA 3 days before vitrectomy in patients with PDR may be effective in decreasing the surgery time and number of iatrogenic retinal breaks. To our knowledge, this is the first report on the co-application of bevacizumab and t-PA to improve the surgical outcomes of vitrectomy in patients with PDR. Our results support further examinations of this approach in a larger series.
